# The well-developed actin cytoskeleton and Cthrc1 expression by actin-binding protein drebrin in myofibroblasts promote cardiac and hepatic fibrosis

**DOI:** 10.1016/j.jbc.2023.102934

**Published:** 2023-01-20

**Authors:** Takanori Hironaka, Noburo Takizawa, Yuto Yamauchi, Yuma Horii, Michio Nakaya

**Affiliations:** Department of Disease Control, Graduate School of Pharmaceutical Sciences, Kyushu University, Fukuoka, Japan

**Keywords:** fibrosis, myofibroblast, hepatic stellate cell (HSC), actin, cytoskeleton, α-SMA, α-smooth muscle actin, BSA, bovine serum albumin, CCl_4_, carbon tetrachloride, CDAHFD, Choline-deficient, L-amino acid-defined, high-fat diet, Cthrc1, collagen triple helix repeat containing 1, DAVID, Database for Annotation, Visualization and Integrated Discovery, drebrin, developmentary regulated brain protein, DMEM, Dulbecco’s Modified Eagle’s Medium, ECM, extracellular matrix, FBS, fetal bovine serum, HSC, Hepatic stellate cell, KEGG, Kyoto Encyclopedia of Genes and Genomes, MRTF, myocardin-related transcription factor, MI, myocardial infarction, NASH, nonalcoholic steatohepatitis, PFA, paraformaldehyde, SEM, standard error of mean, SRF, serum response factor, SOX9, SRY-Box Transcription Factor 9, TGF, transforming growth factor, TPM, transcript per million

## Abstract

Fibrosis is mainly triggered by inflammation in various tissues, such as heart and liver tissues, and eventually leads to their subsequent dysfunction. Fibrosis is characterized by the excessive accumulation of extracellular matrix proteins (*e.g.,* collagens) produced by myofibroblasts. The well-developed actin cytoskeleton of myofibroblasts, one of the main features differentiating them from resident fibroblasts in tissues under inflammatory conditions, contributes to maintaining their ability to produce excessive extracellular matrix proteins. However, the molecular mechanisms *via* which the actin cytoskeleton promotes the production of fibrosis-related genes in myofibroblasts remain unclear. In this study, we found, *via* single-cell analysis, that developmentally regulated brain protein (drebrin), an actin-binding protein, was specifically expressed in cardiac myofibroblasts with a well-developed actin cytoskeleton in fibrotic hearts. Moreover, our immunocytochemistry analysis revealed that drebrin promoted actin cytoskeleton formation and myocardin-related transcription factor–serum response factor signaling. Comprehensive single-cell analysis and RNA-Seq revealed that the expression of collagen triple helix repeat containing 1 (Cthrc1), a fibrosis-promoting secreted protein, was regulated by drebrin in cardiac myofibroblasts *via* myocardin-related transcription factor–serum response factor signaling. Furthermore, we observed the profibrotic effects of drebrin exerted *via* actin cytoskeleton formation and the *Cthrc1* expression regulation by drebrin in liver myofibroblasts (hepatic stellate cells). Importantly, RNA-Seq demonstrated that drebrin expression levels increased in human fibrotic heart and liver tissues. In summary, our results indicated that the well-developed actin cytoskeleton and *Cthrc1* expression due to drebrin in myofibroblasts promoted cardiac and hepatic fibrosis, suggesting that drebrin is a therapeutic target molecule for fibrosis.

Fibrosis is an essential physiological response involved in tissue repair in various organs; however, excessive fibrosis leads to tissue dysfunction due to extracellular matrix (ECM) protein accumulation (1, 2, 3). For example, in the initial myocardial infarction (MI) stages, fibrosis is aggressively induced in the infarct area to prevent cardiac rupture (4, 5, 6). However, in hearts with chronic inflammation after MI, excessive ECM accumulation causes cardiac plasticity loss, thereby worsening cardiac functions (4, 5, 6). Thus, fibrosis contributes to cardiovascular diseases, the leading cause of death worldwide (7). In addition to cardiac fibrosis, hepatic fibrosis is also currently receiving increasing attention. Nonalcoholic steatohepatitis (NASH) is one of the leading diseases associated with hepatic fibrosis and is estimated to affect 3 to 6% of the U.S. population (8, 9). In NASH, a chronic inflammatory disease, fibrosis progresses slowly, unlike in MI, which transitions from acute to chronic inflammation. Hepatic fibrosis is classified as F0–F4 according to the degree of fibrosis (10). At the F4 stage of cirrhosis, hepatocellular carcinoma risk considerably increases (11). Fibrosis is known to be involved in various diseases in different tissues; however, effective agents for ameliorating fibrosis have not yet been identified (12).

Myofibroblasts are responsible for fibrosis development *via* ECM production (3, 13). These cells have a well-developed actin cytoskeleton and are therefore characterized by α-smooth muscle actin (α-SMA) expression; this well-developed actin cytoskeleton also contributes to ECM production (13, 14, 15). Previous studies have shown that myocardin-related transcription factor (MRTF)–serum response factor (SRF) signaling greatly contributes to the positive feedback loop of ECM production depending on actin cytoskeleton formation, because the signaling is activated by actin polymerization induced by mechanical stimuli from the ECMs and in turn promotes the transcription of ECM proteins, including collagens, and genes involved in actin cytoskeleton formation, such as *Acta2* encoding α-SMA (15, 16, 17). However, the molecules responsible for the formation of the well-developed actin cytoskeleton that characterizes myofibroblasts remain largely unidentified.

Developmentally regulated brain protein (drebrin) was first identified in the chicken optic tectum (18); it has been reported to play important roles by binding to F-actin and its stability (19). The role of drebrin in the actin cytoskeleton has been studied in brain cells (20, 21, 22) but remains unclear for other cell types. We previously found that drebrin, expressed in cardiac and lung myofibroblasts, promotes fibrosis-related gene expression (23). However, the molecular mechanisms underlying the fibrosis-promoting effects of drebrin remain unclear.

Collagen triple helix repeat containing 1 (Cthrc1) was first identified as a protein whose expression is highly induced in arteries following balloon injury (24). Previous studies have reported that Cthrc1 is involved in tumorigenesis, proliferation, and metastasis in various human malignancies because it regulates diverse signaling pathways such as the transforming growth factor (TGF)-β signaling pathway (25). Recent single-cell transcriptomic studies have proposed Cthrc1 as a new marker for myofibroblasts, which play a crucial role in fibrosis development in the heart and lungs (26, 27). In addition, Cthrc1 promotes cardiac fibrosis (26) and carbon tetrachloride (CCl_4_)- and thioacetamide-induced hepatic fibrosis (28); however, the mechanism underlying Cthrc1 expression remains unclear.

In this study, we found that drebrin promoted fibrosis by inducing MRTF–SRF signaling *via* actin cytoskeleton formation in myofibroblasts and by increasing their Cthrc1 expression in fibrotic heart and liver tissues.

## Results

### Single-cell analysis revealed unique phenotypes of drebrin-expressing fibroblasts

**Figure 1:**
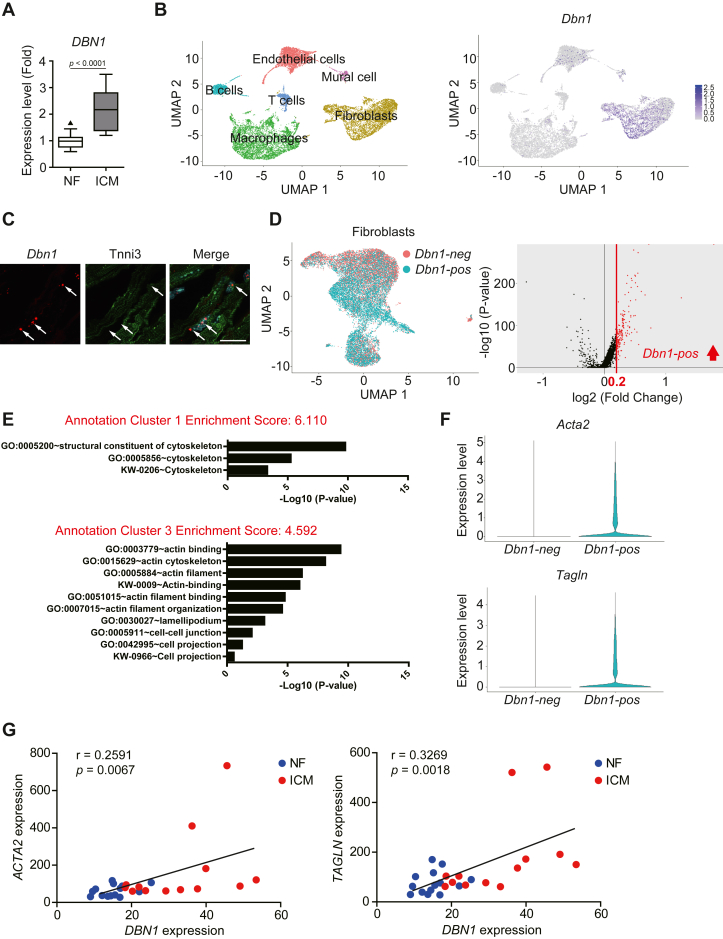
***Dbn1* expression increases in myofibroblasts with well-developed actin cytoskeleton in fibrotic hearts**. *A*, *DBN1* mRNA expression levels in nonfailing (NF) or ischemic (ICM) human hearts. Publicly available data (GSE116250) were reanalyzed. NF: n = 14; ICM: n = 13. *B*, UMAP plots and *Dbn1* mRNA expression levels in mouse cardiac interstitial cells 3 or 7 days after sham operation or MI. The publicly available data (E-MTAB-7376) were reanalyzed using the R package Seurat. *C*, *Dbn1* mRNA expression in mouse fibrotic hearts. The heart sections obtained 7 days after MI were subjected to *in situ* hybridization for *Dbn1* and immunohistochemistry with an anti-Tnni3 antibody. *White arrows* indicate the *Dbn1* signals. Scale bar: 20 μm. *D*, plot of *Dbn1*-positive (*Dbn1*-pos) or *Dbn1*-negative (*Dbn1*-neg) fibroblasts and volcano plot of genes differentially expressed between *Dbn1*-positive and *Dbn1*-negative fibroblasts. The publicly available data (E-MTAB-7376) were reanalyzed using the R package Seurat. The plots divided into *Dbn1*-positive and *Dbn1*-negative fibroblasts are shown in the left panel. The volcano plot of genes differentially expressed between *Dbn1*-positive and *Dbn1*-negative fibroblasts is shown in the right panel. The x-axis represents fold change between the two groups, and the y-axis represents the *p*-value. The red line represents 0.2-fold increase. *E*, annotation cluster of genes differentially expressed between *Dbn1*-positive and *Dbn1*-negative fibroblasts. The differentially expressed genes were analyzed using DAVID; enriched annotation clusters are shown. *F*, *Acta2* and *Tagln* mRNA expression levels of *Dbn1*-positive and *Dbn1*-negative fibroblasts. The violin plots between *Dbn1*-positive and *Dbn1*-negative fibroblasts were drawn using the data reanalyzed in Figure 1*D*. *G*, relative *DBN1* and *Acta2* or *TAGLN* mRNA expression in NF or ICM human hearts. The publicly available data (GSE116250) were reanalyzed. The x-axis represents *DBN1* expression, and the y-axis represents *Acta2* (*left panel*) or *TAGLN* (*right panel*) expression. The *blue* and *red* plots indicate the NF and ICM human hearts, respectively. NF: n = 14; ICM: n = 13. DAVID, Database for Annotation, Visualization and Integrated Discovery; MI, myocardial infarction

We previously showed that drebrin expression is induced in fibrotic mouse hearts and lungs and promotes the expression of fibrosis-related genes in myofibroblasts in these tissues (23). In the current study, using the datasets of human patients with idiopathic cardiomyopathy (GSE116250) (29), we found that drebrin was also induced in human fibrotic hearts (Fig. 1*A*), suggesting that it also contributed to human cardiac fibrosis. However, the role of drebrin in fibrotic tissues remains unclear. To clarify its role, we first performed single-cell analysis of stromal cells (cardiac cells other than cardiomyocytes) in mouse hearts after MI by using publicly available datasets (E-MTAB-7376) (30). Cardiac stromal cells were divided into six clusters on the basis of expression levels of marker proteins for each cell population (Fig. 1*B*): fibroblasts (*e.g.*, *Col1a1*), endothelial cells (*e.g.*, *Kdr*), macrophages (*e.g*., *Cd68*), B cells (*e.g.*, *Cd79a*), T cells (*e.g*., *Cd3d*), and mural cells (*e.g*., *Vtn*) (Fig. S1). Drebrin was mainly expressed in the fibroblast cluster containing myofibroblasts (Fig. 1*B*). We also confirmed that cardiomyocytes did not express drebrin in fibrotic hearts (Fig. 1*C*). To further investigate the roles of drebrin in fibrotic hearts, we analyzed another dataset focusing on whole fibroblasts (GFP-Pdgfra positive and CD31 negative cells from E-MTAB-7376) containing myofibroblasts. The fibroblasts were divided into two groups on the basis of drebrin expression: *Dbn1*-positive and *Dbn1*-negative cells (Fig. 1*D*). We then compared the gene expression in the two-cell population; the volcano plot showed that many genes showed high fold-change in expression between the two groups (Fig. 1*D*). We then compared the gene expression in the two-cell population. We performed a Database for Annotation, Visualization and Integrated Discovery (DAVID) analysis of the genes showing abundant expression in *Dbn1*-positive cells and found that actin cytoskeleton-related genes were enriched in *Dbn1*-positive cells (Fig. 1*E*). Among their gene products, α-SMA encoded by *Acta2* and SM22α encoded by *Tagln*, both of which are myofibroblast markers that play important roles in actin cytoskeleton formation, were abundant in *Dbn1*-positive cells (Fig. 1*F*). Consistent with this result, the *DBN1* expression level correlated with *Acta2* and *TAGLN* levels in the hearts of patients with idiopathic cardiomyopathy (Fig. 1*G*). These results indicate that drebrin is expressed in myofibroblasts with a well-developed actin cytoskeleton in fibrotic hearts.

### Drebrin promoted actin cytoskeleton formation in cardiac myofibroblasts

**Figure 2:**
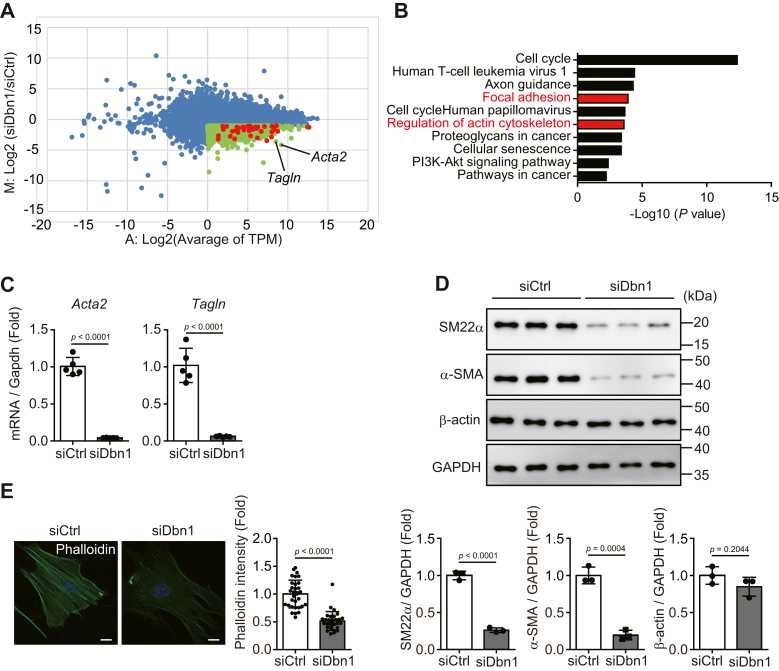
**Drebrin contributes to actin cytoskeleton formation in cardiac myofibroblasts**. *A*, MA plot of genes differentially expressed between siCtrl and siDbn1 myofibroblasts. At 72 h after siRNA transfection, the cardiac myofibroblasts were lysed and subjected to RNA-Seq, and the MA plots were drawn using the TPM values obtained. The x-axis represents the TPM value for gene expression, and the y-axis represents fold change between the two groups. The *green plots* indicate the genes downregulated on silencing *Dbn1* (M < −1.0, A > 0), and the *red plots* indicate the genes in GO terms associated with focal adhesion and regulation of the actin cytoskeleton. *B*, Kyoto Encyclopedia of Genes and Genomes (KEGG) pathway enrichment analysis of genes differentially expressed between the two groups. The genes downregulated on silencing *Dbn1* (M < −1.0, A > 0) were analyzed using DAVID; the enriched KEGG pathways (gene count > 25) are shown. *C*, *Acta2* or *Tagln* mRNA expression in cardiac myofibroblasts treated with siCtrl or siDbn1. At 72 h after siRNA transfection, the cardiac myofibroblasts were lysed and subjected to qRT-PCR. siCtrl: n = 5; siDbn1: n = 5. *D*, α-SMA, SM22α, or β-actin protein levels in cardiac myofibroblasts treated with siCtrl or siDbn1. At 60 h after siRNA transfection, the cardiac myofibroblasts were starved for 12 h, lysed, and subjected to immunoblot analysis. The panel below shows quantification of α-SMA, SM22α, or β-actin expression. *E*, phalloidin staining of F-actin in cardiac myofibroblasts treated with siCtrl or siDbn1. At 48 h after siRNA transfection, the cardiac myofibroblasts were seeded onto glass-bottom dishes, cultured for 24 h and subjected to phalloidin staining. Scale bar: 20 μm. The *right panel* shows quantification of phalloidin intensity. siCtrl: n = 32; siDbn1: n = 30. DAVID, Database for Annotation, Visualization and Integrated Discovery; TPM, transcript per million; α-SMA, α-smooth muscle actin.

To investigate the contribution of drebrin to cardiac fibrosis *in vitro*, we isolated cardiac myofibroblasts from infarcted mouse hearts 3 days after MI and cultured them for 2 days on plastic plates. Flow cytometry analysis of the cultured cells showed that almost all cells expressed α-SMA (Fig. S2), indicating that they are myofibroblasts. Then, we treated these cardiac myofibroblasts with siRNA against *Dbn1* and performed RNA-Seq (Fig. 2*A*). The genes whose expression decreased on *Dbn1* knockdown were subjected to Kyoto Encyclopedia of Genes and Genomes (KEGG) pathway analysis; drebrin was found to be highly involved in the mRNA expression of molecules related to the actin cytoskeleton and focal adhesion, such as *Acta2* and *Tagln* (Fig. 2, *A* and *B*). Using qRT-PCR analysis, we confirmed that *Dbn1* knockdown markedly decreased *Acta2* and *Tagln* mRNA expression (Fig. 2*C*). Consistent with these findings, *Dbn1* knockdown also decreased α-SMA and SM22α protein levels in cardiac myofibroblasts (Fig. 2*D*). On the other hand, *Dbn1* knockdown did not affect the protein level of β-actin, which is one of the cytoplasmic actins, in cardiac myofibroblasts (Fig. 2*D*), suggesting that drebrin specifically contributes to the actin cytoskeleton formation by α-SMA and SM22α, both of which are characteristic proteins of myofibroblasts. We further investigated the effect of drebrin on actin cytoskeleton formation by using phalloidin staining, which specifically detects F-actin. We found that F-actin cytoskeleton formation was considerably suppressed by *Dbn1* knockdown in cardiac myofibroblasts (Fig. 2*E*). These results showed that drebrin promoted the expression of genes involved in actin cytoskeleton formation, such as α-SMA and SM22α, in cardiac myofibroblasts.

### Drebrin promoted myofibroblast differentiation by enhancing actin–MRTF–SRF signaling

**Figure 3:**
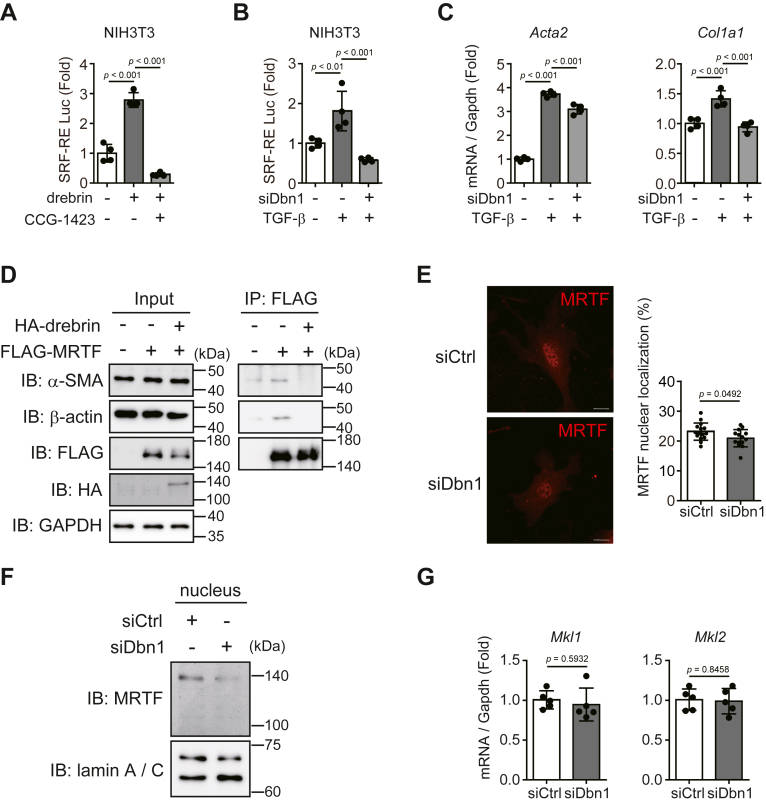
**Drebrin promotes MRTF–SRF signaling**. *A*, luminescence obtained using the SRF-RE reporter in drebrin-overexpressing NIH3T3 cells after CCG-1423 treatment. At 24 h after plasmid transfection, the NIH3T3 cells were starved for 24 h, treated with CCG-1423 (10 μM) for 24 h, lysed, and subjected to qRT-PCR. n = 4. *B*, luminescence obtained using the SRF-RE reporter in *Dbn1*-knockdown NIH3T3 cells after TGF-β stimulation. At 48 h after siRNA transfection, the NIH3T3 cells were stimulated with TGF-β (2 ng/ml) for 24 h, lysed, and subjected to the luciferase assay. n = 4. *C*, *Acta2* and *Col1a1* mRNA expression levels in NIH3T3 cells treated with siCtrl or siDbn1 after TGF-β stimulation. At 48 h after siRNA transfection, the NIH3T3 cells were stimulated with TGF-β (2 ng/ml) for 24 h, lysed, and subjected to qRT-PCR. n = 4. *D*, binding between MRTF and α-SMA or β-actin in drebrin-overexpressing NIH3T3 cells. At 48 h after plasmid transfection, the NIH3T3 cells were lysed and subjected to the immunoprecipitation assay. n = 3. *E*, MRTF translocation in cardiac myofibroblasts treated with siCtrl or siDbn1. At 48 h after siRNA transfection, the cells were seeded onto glass-bottom dishes, cultured for 24 h, and subjected to immunocytochemistry. Scale bar: 20 μm. The *right panel* shows quantification of MRTF intensity. siCtrl; n = 15, siDbn1; n = 14. *F*, MRTF protein expressions in nuclear extraction of cardiac myofibroblasts treated with siCtrl or siDbn1. At 72 h after siRNA transfection, the cardiac myofibroblasts were lysed and subjected to immunoblot analysis. n = 3. *G*, *Mkl1* or *Mkl2* mRNA expression in cardiac myofibroblasts treated with siCtrl or siDbn1. At 72 h after siRNA transfection, the cardiac myofibroblasts were lysed and subjected to qRT-PCR. siCtrl: n = 5; siDbn1: n = 5. MRTF, myocardin-related transcription factor; SRF, serum response factor; α-SMA, α-smooth muscle actin; TGF, transforming growth factor.

MRTF–SRF signaling is an important pathway involved in myofibroblast differentiation and *Acta2* transcription (15, 16). Therefore, we investigated whether drebrin contributes to activating MRTF–SRF signaling. We overexpressed drebrin in NIH3T3 cells, a mouse fibroblast cell line, and performed a luciferase assay by using SRF-RE Luc, which specifically detects MRTF–SRF signaling. Drebrin overexpression extensively enhanced MRTF–SRF signaling in NIH3T3 cells; this enhancement was counteracted by treatment with CCG-1423, an MRTF–SRF pathway inhibitor (Fig. 3*A*).

NIH3T3 cells have been previously found to acquire a myofibroblast-like phenotype upon TGF-β stimulation *via* SRF activation, leading to increase in their ability to produce collagens and other fibrotic factors (31). Therefore, we further investigated whether drebrin is involved in TGF-β/MRTF-SRF–mediated differentiation of NIH3T3 cells into myofibroblast-like cells. The SRF-RE Luc assay showed that TGF-β stimulation activated the MRTF–SRF pathway, which was markedly attenuated by *Dbn1* knockdown (Fig. 3*B*). Consistent with these data, *Col1a1* and *Acta2* upregulation due to TGF-β stimulation in NIH3T3 cells was notably suppressed by *Dbn1* knockdown (Fig. 3*C*).

MRTF, a transcription cofactor, is located in the cytoplasm and interacts with G-actin. When G-actin is polymerized to F-actin, MRTF dissociates from G-actin and translocates into the nucleus, promoting the transcription of fibrosis-related genes, such as *Acta2* and collagens. Because drebrin is an actin-binding protein, we tested whether it affects the binding of MRTF to α-SMA or β-actin, which is one of the major cytoplasmic actins. Immunoprecipitation analysis showed that the amounts of α-SMA or β-actin binding to FLAG-MRTF decreased when HA-drebrin was overexpressed (Fig. 3*D*). We further found, *via* immunocytochemical analysis, that *Dbn1* knockdown decreased MRTF nuclear localization in cardiac myofibroblasts (Figs. 3*E* and S3). The decrease in the amount of nuclear MRTF by *Dbn1* knockdown in the cells was also confirmed by Western blotting (Fig. 3*F*). On the contrary, drebrin did not affect the expression of MRTF (Fig. 3*G*), indicating that the promotion of MRTF–SRF signaling by drebrin is not dependent on the expression level of MRTF, but on the regulation of MRTF nuclear translocation.

These results showed that drebrin promoted MRTF–SRF signaling by regulating actin polymerization.

### Drebrin increased the expression of the profibrotic gene Cthrc1

**Figure 4:**
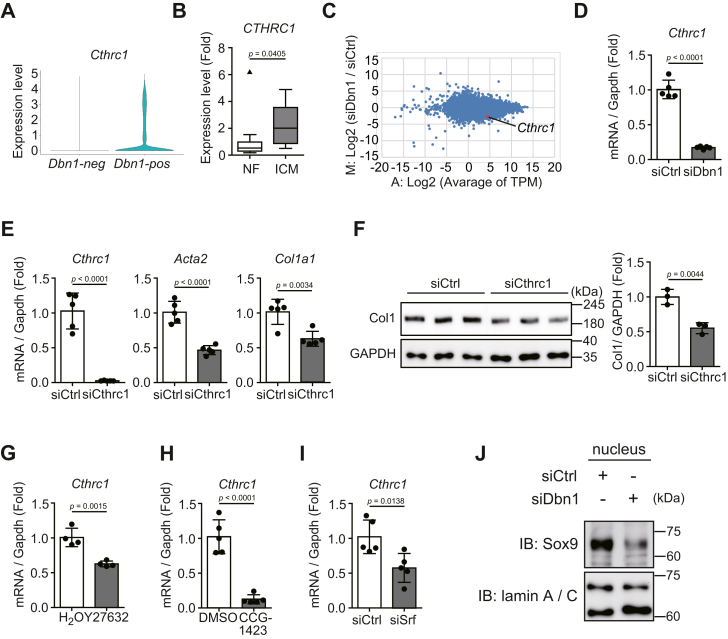
**Drebrin promotes the Cthrc1 expression *via* MRTF–SRF mediated signaling**. *A*, *Cthrc1* mRNA expression levels of *Dbn1*-positive and *Dbn1*-negative fibroblasts. The violin plots between *Dbn1*-positive and *Dbn1*-negative fibroblasts were drawn by using the data reanalyzed in Fig.1*D*. *B*, *CTHRC1* mRNA expression levels in nonfailing (NF) or ischemic (ICM) human hearts. The publicly available data (GSE116250) were reanalyzed. NF: n = 14; ICM: n = 13. *C*, MA plot of genes differentially expressed between siCtrl and siDbn1 myofibroblasts. At 72 h after siRNA transfection, the cardiac myofibroblasts were lysed and subjected to RNA-Seq, and MA plots were drawn using the TPM values obtained. The x-axis represents the TPM value for gene expression, and the y-axis represents fold change between the two groups. *D*, *Cthrc1* mRNA expression levels in cardiac myofibroblasts treated with siCtrl or siDbn1. At 72 h after siRNA transfection, the cardiac myofibroblasts were lysed and subjected to qRT-PCR. n = 5. *E*, the mRNA expression levels of fibrosis-related genes in cardiac myofibroblasts treated with siCtrl or siCthrc1. At 72 h after siRNA transfection, the cardiac myofibroblasts were lysed and subjected to qRT-PCR. n = 5. *F*, collagen1a1 (Col1) protein levels in cardiac myofibroblasts treated with siCtrl or siCthrc1. At 72 h after siRNA transfection, the cardiac myofibroblasts were lysed and subjected to immunoblot analysis. The *right panel* shows quantification of Collagen1a1 expression. n = 3. *G*, *Cthrc1* mRNA expression levels in cardiac myofibroblasts treated with H_2_O or Y27632. Cardiac myofibroblasts were treated with Y27632 (30 μM) for 24 h, lysed, and subjected to qRT-PCR. n = 4. *H*, *Cthrc1* mRNA expression levels in cardiac myofibroblasts treated with DMSO or CCG-1423. Cardiac myofibroblasts were starved for 24 h, treated with CCG-1423 (10 μM) for 24 h, lysed, and subjected to qRT-PCR. n = 5. *I*, *Cthrc1* mRNA expression levels in cardiac myofibroblasts treated with siCtrl or siSRF. At 72 h after siRNA transfection, the cardiac myofibroblasts were lysed and subjected to qRT-PCR. n = 5. *J*, SOX9 protein expressions in nuclear extraction of cardiac myofibroblasts treated with siCtrl or siDbn1. At 72 h after siRNA transfection, the cardiac myofibroblasts were lysed and subjected to immunoblot analysis. n = 3. MRTF, myocardin-related transcription factor; SRF, serum response factor; Cthrc1, collagen triple helix repeat containing 1; TPM, transcript per million.

Our findings suggested that drebrin regulated myofibroblast differentiation by activating MRTF–SRF signaling. Therefore, we aimed to identify the profibrotic molecules regulated by *Dbn1* expression. We reanalyzed the single-cell data (Fig. 1*D*) and found that *Cthrc1*, a secreted protein, was specifically expressed in *Dbn1*-positive cells (Fig. 4*A*). Consistent with this result, our analysis of datasets of patients with idiopathic cardiomyopathy (GSE116250) confirmed that *CTHRC1* expression increased in human fibrotic hearts (Fig. 4*B*). Furthermore, we reanalyzed RNA-seq data (Fig. 2*A*) and found that *Cthrc1* was downregulated *via Dbn1* knockdown (Fig. 4*C*) in cardiac myofibroblasts. This decrease in *Cthrc1* expression following *Dbn1* knockdown was confirmed by qRT-PCR (Fig. 4*D*).

Cthrc1 has been reported to increase the levels of fibrosis-related proteins, such as collagens, and recent single-cell studies identified Cthrc1 as a new marker protein for myofibroblasts (26, 27). As previously reported (26), qRT-PCR analysis and Western blotting showed that Cthrc1 promoted the expression of fibrosis-related genes in cardiac myofibroblasts (Fig. 4, *E* and *F*).

Drebrin stabilizes F-actin (19); therefore, we considered that F-actin stabilization was important for Cthrc1 expression. Cardiac myofibroblast treatment with Y27632, an inhibitor of Rho-associated coiled-coil kinase, which stabilizes F-actin by activating LIM kinase that was responsible for cofilin inactivation, considerably suppressed Cthrc1 expression, indicating that F-actin stabilization is important for Cthrc1 expression (Fig. 4*G*). We then examined the mechanism by which drebrin promotes Cthrc1 expression. Given that drebrin promotes MRTF–SRF signaling, we hypothesized that the Cthrc1 expression level may depend on this signaling. Therefore, we treated cardiac myofibroblasts with CCG-1423 (Fig. 4*H*), an inhibitor of MRTF-SRF signaling, or siRNA against SRF (Fig. 4*I*). The qRT-PCR assays showed that both CCG-1423 treatment (Fig. 4*H*) and SRF knockdown (Fig. 4*I*) markedly suppressed *Cthrc1* expression. On the other hand, it was reported that Cthrc1 expression was promoted by a transcription factor, SRY-Box transcription factor 9 (SOX9), in cardiac myofibroblasts (26). In addition, the nuclear translocation of SOX9 is attenuated in the scarring astrocytes of drebrin-KO mice (22). Therefore, we hypothesized that drebrin also promotes Cthrc1 expression by regulating the nuclear translocation of SOX9. To examine this, we compared the amount of nuclear SOX9 in myofibroblast treated with siCtrl or siDbn1 (Fig. 4*J*). The result showed that the amount of nuclear SOX9 was markedly reduced by siDbn1 treatment, indicating that drebrin enhances the SOX9 translocation in myofibroblasts (Fig. 4*J*).

These results showed that F-actin stabilization by drebrin regulated *Cthrc1* expression levels in cardiac myofibroblasts *via* MRTF–SRF signaling.

### Drebrin expression was induced during hepatic fibrosis and promoted the expression of fibrosis-related genes in activated hepatic stellate cells

**Figure 5:**
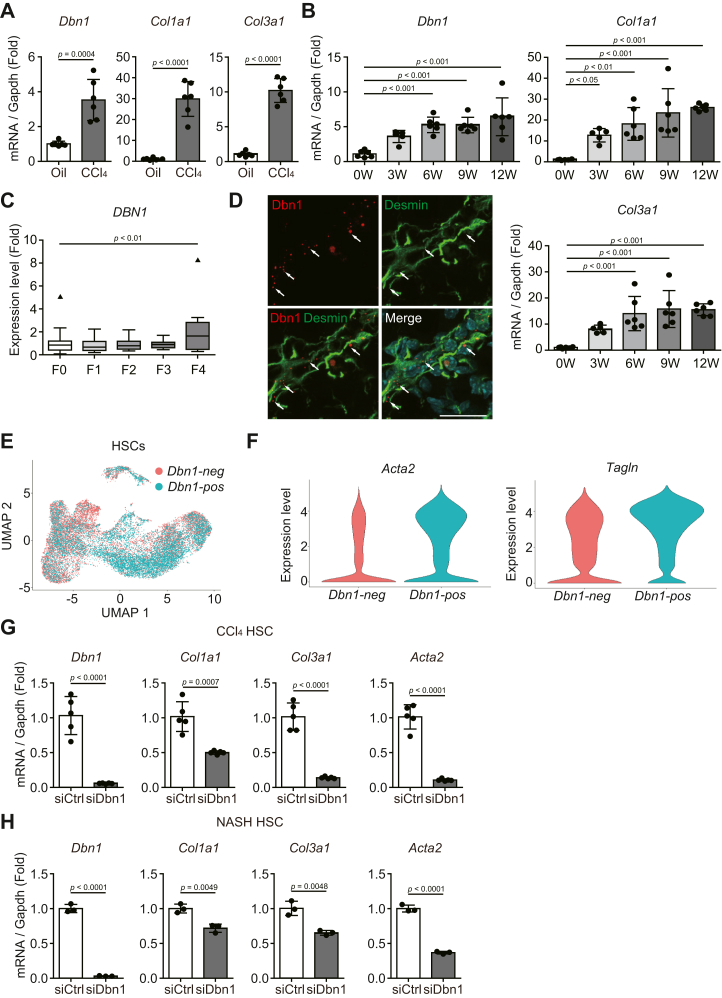
**Drebrin expression is induced during hepatic fibrosis and promotes the expression of fibrosis-related genes in activated HSCs**. *A*, *Dbn1*, *Col1a1*, and *Col3a1* mRNA expression levels in the livers of mice injected with CCl_4_ for 4 weeks. Total RNA extracted from the mouse livers was used for qRT-PCR. n = 6. *B*, *Dbn1*, *Col1a1*, and *Col3a1* mRNA expression levels in the livers of mice fed a CDAHFD for 0, 3, 6, 9, or 12 weeks. Total RNA extracted from the mouse livers was used for qRT-PCR. 0, 6, 9, and 12 weeks: n = 6; 3 weeks: n = 5. *C*, *DBN1* mRNA expression levels in patients with various stages (F0–F4) of hepatic fibrosis. The publicly available data (GSE162694) were reanalyzed. F0: n = 35; F1: n = 30; F2: n = 27; F3: n = 8; F4: n = 12. *D*, *Dbn1* mRNA expression in mouse fibrotic livers. Liver sections from mice fed a CDAHFD for 10 weeks were subjected to *in situ* hybridization and immunohistochemistry with an anti-Desmin antibody. *White arrows* indicate the *Dbn1* signals. Scale bar: 20 μm. *E*, plot of *Dbn1*-positive (*Dbn1*-pos) or *Dbn1*-negative (*Dbn1*-neg) HSCs and volcano plot of genes differentially expressed between *Dbn1*-positive and *Dbn1*-negative HSCs. The publicly available data (GSE171904) were reanalyzed using the R package Seurat, and the plots divided into *Dbn1*-positive and *Dbn1*-negative HSCs are shown. *F*, *Acta2* and *Tagln* mRNA expression levels of *Dbn1*-positive and *Dbn1*-negative HSCs. The violin plots between *Dbn1*-positive and *Dbn1*-negative HSCs were drawn using the data reanalyzed in Figure 5*E*. *G*, mRNA expression levels of fibrosis-related genes in activated HSCs isolated from CCl_4_-treated livers after treatment with siCtrl or siDbn1. At 72 h after siRNA transfection, the activated HSCs isolated from CCl_4_-induced hepatic fibrosis model mice were lysed and subjected to qRT-PCR. n = 5. *H*, mRNA expression levels of fibrosis-related genes in activated HSCs isolated from a CDAHFD-fed livers after treatment with siCtrl or siDbn1. At 72 h after siRNA transfection, the activated HSCs isolated from a CDAHFD-induced NASH model mice were lysed and subjected to qRT-PCR. n = 3. CDAHFD, Choline-deficient, L-amino acid-defined, high-fat diet; HSCs, hepatic stellate cells; CCl_4_, carbon tetrachloride; NASH, nonalcoholic steatohepatitis.

We examined drebrin involvement in hepatic fibrosis by using a mouse model of CCl_4_-induced hepatic fibrosis. CCl_4_ causes hepatocyte death and repeated CCl_4_ administration induces liver injury, leading to fibrosis (32). The qRT-PCR assays showed that *Dbn1*, *Col1a1*, and *Col3a1* expression levels increased during CCl_4_-induced hepatic fibrosis (Fig. 5*A*). We then investigated whether drebrin increased in the liver of a NASH mouse model with fibrosis. To establish a NASH mouse model, the mice were fed a choline-deficient, L-amino acid-defined, high-fat diet (CDAHFD) for 3, 6, 9, or 12 weeks. The temporal CDAHFD-feeding experiment revealed that *Dbn1* expression was induced as the hepatic fibrosis progressed, along with increase in *Col1a1* and *Col3a1* expression (Fig. 5*B*). Consistent with this result, *DBN1* expression was found to increase in patients with advanced stages of fibrosis (GSE162694) (33) (Fig. 5*C*).

We then aimed to determine the types of cells expressing drebrin in the fibrotic liver. *In situ* hybridization assays using fibrotic mouse livers showed that *Dbn1* mRNA was present in Desmin-positive cells, indicating that drebrin was expressed in activated hepatic stellate cells (HSCs), which are the main collagen-producing cells in hepatic fibrosis (34) (Fig. 5*D*). To characterize *Dbn1* expressing cells in fibrotic mouse livers, we performed single-cell analysis of HSCs in mouse livers injected with corn oil or CCl_4_ by using publicly available datasets (GSE171904) (35). We divided HSCs into *Dbn1*-positive and *Dbn1*-negative cells (Fig. 5*E*) and compared the expression levels of the genes involved in actin cytoskeleton formation; our analyses showed high *Acta2* and *Tagln* expression levels in *Dbn1*-positive cells (Fig. 5*F*).

We investigated whether drebrin promotes the expression of fibrosis-related genes in activated HSCs. The qRT-PCR analyses revealed that *Dbn1* knockdown extensively decreased their expression in activated HSCs isolated from CCl_4_-induced or CDAHFD-induced fibrotic mouse livers (Fig. 5, *G* and *H*). Furthermore, this decrease in the expression of fibrosis-related genes following *DBN1* knockdown was also observed in the LX-2 human HSC cell line (Fig. S4).

These results showed that drebrin expression was induced during hepatic fibrosis and promoted the expression of fibrosis-related genes *via* actin cytoskeleton formation in activated HSCs.

### Regulation of Cthrc1 expression *via* the drebrin-MRTF–SRF axis was also observed in activated HSCs

**Figure 6:**
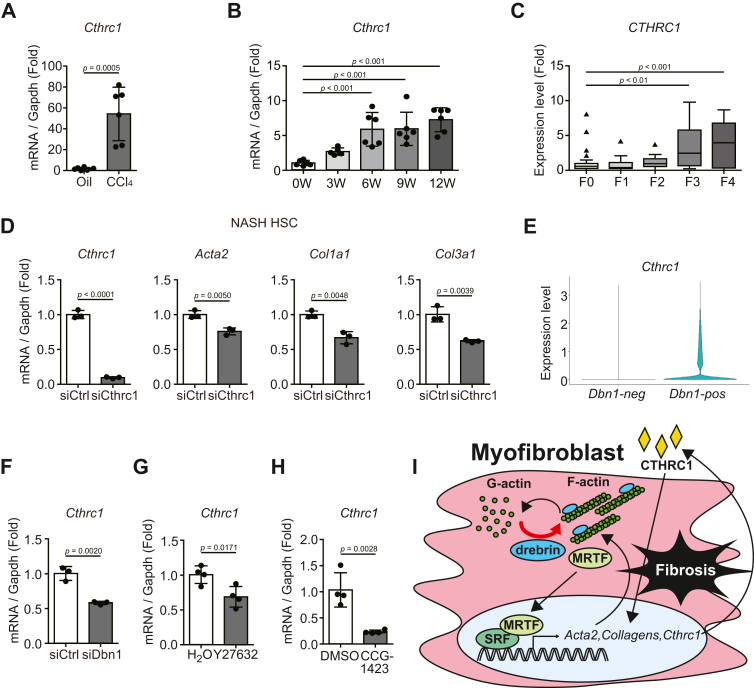
**Regulation of Cthrc1 expression levels *via* the drebrin-MRTF-SRF axis is also observed in activated HSCs**. *A*, *Cthrc1* mRNA expression levels in the livers of mice injected with CCl_4_ for 4 weeks. Total RNA extracted from the mouse livers was subjected to qRT-PCR. n = 6. *B*, *Cthrc1* mRNA expression levels in the livers of mice fed a CDAHFD for 0, 3, 6, 9, or 12 weeks. Total RNA extracted from the mouse livers was subjected to qRT-PCR. 0, 6, 9, and 12 weeks: n = 6; 3 weeks: n = 5. *C*, *CTHRC1* mRNA expression levels of patients with various stages (F0–F4) of hepatic fibrosis. The publicly available data (GSE162694) were reanalyzed. F0: n = 35; F1: n = 30; F2: n = 27; F3: n = 8; F4: n = 12. *D*, the mRNA expression levels of fibrosis-related genes in activated HSCs treated with siCtrl or siCthrc1. At 72 h after siRNA transfection, the activated HSCs isolated from a CDAHFD-induced NASH model mice were lysed and subjected to qRT-PCR. n = 3. *E*, *Cthrc1* mRNA expression levels of *Dbn1*-positive and *Dbn1*-negative HSCs. The violin plots between *Dbn1*-positive and *Dbn1*-negative HSCs were drawn using the data reanalyzed in Figure 5*E*. *F*, *Cthrc1* mRNA expression levels in activated HSCs isolated from a CDAHFD-fed livers after treatment with siCtrl or siDbn1. At 72 h after siRNA transfection, the activated HSCs isolated from a CDAHFD-induced NASH model mice were lysed and subjected to qRT-PCR. n = 3. *G*, *Cthrc1* mRNA expression levels in activated HSCs isolated from CCl_4_-treated livers after treatment with H_2_O or Y27632. Activated HSCs isolated from CCl_4_-induced hepatic fibrosis model mice were treated with Y27632 (30 μM) for 24 h, lysed, and subjected to qRT-PCR. n = 4. *H*, *Cthrc1* mRNA expression levels in activated HSCs treated with DMSO or CCG-1423. Activated HSCs isolated from a CDAHFD-induced NASH model mice were starved for 24 h, treated with CCG-1423 (10 μM) for 24 h, lysed, and subjected to qRT-PCR. n = 4. *I*, graphical abstract of the fibrosis-promoting mechanism of drebrin involving actin cytoskeleton formation in myofibroblasts. CDAHFD, choline-deficient, L-amino acid-defined, high-fat diet; HSCs, hepatic stellate cells; CCl_4_, carbon tetrachloride; NASH, nonalcoholic steatohepatitis; Cthrc1, collagen triple helix repeat containing 1.

We examined the effect of drebrin on Cthrc1 expression in activated HSCs. Increased Cthrc1 expression has been previously detected in the fibrotic livers of CCl_4_- or thioacetamide-administered mice, and autocrine Cthrc1 has been found to activate HSCs and promote hepatic fibrosis (28). We confirmed that *Cthrc1* expression increased during CCl_4_-induced hepatic fibrosis (Fig. 6*A*). Furthermore, we found that *Cthrc1* expression gradually increased in CDAHFD-treated NASH fibrosis mouse models (Fig. 6*B*). Consistent with these data, *CTHRC1* expression was found to increase in the livers of human patients with NASH as fibrosis progressed (Fig. 6*C*).

We then knocked down *Cthrc1* in activated HSCs isolated from CDAHFD-induced NASH mice. The qRT-PCR analysis showed that *Cthrc1* knockdown markedly decreased the expression of fibrosis-related genes, such as *Acta2*, *Col1a1*, *and Col3a1*, in activated HSCs, indicating that Cthrc1 promoted the expression of these genes (Fig. 6*D*).

We investigated whether Cthrc1 expression was regulated by drebrin in fibrotic livers. The violin plot between *Dbn1*-positive and *Dbn1*-negative cells obtained on analysis of the datasets (GSE171904) showed that Cthrc1 was abundant in *Dbn1*-positive cells (Fig. 6*E*). Similar to the results obtained for cardiac myofibroblasts (Fig. 4, *D*, *G* and *H*), treatment of activated HSCs with siRNA against *Dbn1*, Y27632, or CCG-1423 considerably decreased *Cthrc1* expression in activated HSCs (Fig. 6, *F*, *G* and *H*).

These results indicated that F-actin stabilization by drebrin also promoted Cthrc1 expression *via* MRTF–SRF signaling in activated HSCs.

## Discussion

In this study, we found that drebrin stabilized F-actin and promoted collagen production by increasing the level of the fibrosis-promoting secreted protein Cthrc1 in myofibroblasts (Fig. 6*I*). Drebrin plays an important role in dendritic spine and synapse formation in neurons (20) and in maintenance of astrocyte reactivity (22); however, the role of drebrin in cells other than brain cells remains unclear. In this study, we found that drebrin was upregulated during differentiation of fibroblasts into myofibroblasts and that drebrin promoted collagen production in myofibroblasts.

A well-developed actin cytoskeleton is one of the characteristics of myofibroblasts and is thought to play an important role in maintaining their differentiation state and in the production of ECM proteins such as collagen (15, 17). G protein–coupled receptors activation on integrin stimulation by ECM proteins, humoral factors or physical stimuli activates the Rho-associated coiled-coil kinase pathway and stabilizes F-actin in myofibroblasts (16, 36). When F-actin is stabilized, MRTF–SRF signaling is enhanced, and the transcription of molecules important for actin cytoskeleton formation, such as *Acta2*, or the levels of ECM proteins increase. Hence, there is a positive feedback of actin cytoskeleton formation and ECM production. Mice with MRTF-A deletion, which is critical for this feedback, have been found to show lesser cardiac fibrosis than mice without this deletion after MI (37). However, the molecules that directly bind to and stabilize F-actin in myofibroblasts remain unclear. In this study, we demonstrated for the first time that drebrin strongly contributed to F-actin stabilization in myofibroblasts (Fig. 6*I*).

Drebrin plays an important role in dendritic spine formation in neurons by causing the accumulation of postsynaptic proteins such as PSD-95 (38). This PSD-95 gathering by drebrin is supposed to contribute to the efficient reception of neurotransmitters from the presynaptic area. Although synapse-like formation by myofibroblasts have not yet been reported, myofibroblasts were found to intracellularly interact with macrophages *via* homotypic cadherin-11 interactions (39). Thus, drebrin may contribute to the interactions by forming an actin cytoskeleton that serves as a scaffold. In this context, it will be interesting to examine the localization of drebrin in myofibroblasts upon contacting macrophages.

Drebrin is not highly expressed in normal tissues other than the brain and is prominently expressed in the myofibroblasts of fibrotic tissues. In addition, its expression is upregulated in the human heart and liver, in association with fibrosis. Therefore, drebrin may be an attractive target molecule for use in therapeutic agent development for tissue fibrosis. Furthermore, a previous study reported that drebrin KO mice are not embryonic lethal and show no significant defects in brain development (40). Thus, future studies using drebrin KO mice will provide further insight into the contribution of drebrin to fibrosis of various tissues.

## Experimental procedures

### Animal model

Male C57BL/6J (age, 6–10 weeks) were purchased from Japan SLC (Japan) and housed in groups of five mice per cage under a 12:12 h light-dark cycle and appropriate temperature and humidity conditions. All animal experiments and protocols were approved by the Animal Care and Use Committee of Kyushu University (Japan).

For the creation of MI mouse model, anesthetized male mice (age, 8–10 weeks) underwent ligation of the left anterior descending artery.

For the creation of CCl_4_-induced hepatic fibrosis mouse model, male mice (age, 8 weeks) were intraperitoneally administered 100 μl 20% CCl_4_ (Sigma Aldrich) in corn oil (Sigma Aldrich) twice a week for 4 weeks. The control group mice were intraperitoneally administered the same amount of corn oil for the same period.

For the creation of CDAHFD-induced NASH mouse model, male mice (age, 6 weeks) were singly housed and fed a CDAHFD (Research Diets Inc.) for 3, 6, 9, or 12 weeks.

### Isolation of myofibroblasts or activated HSCs from fibrotic tissues by magnetic-activated cell sorting

Cardiac myofibroblasts were isolated as previously described (23). Briefly, the fibrotic hearts of mice 3 days after MI were collected and digested in an enzyme solution, and the cells isolated were seeded onto a culture plate after removing red blood cells. The culture medium was then changed after 6 h, and the cells attached to the plates were collected the next day. These cells were then incubated with anti-CD45 microbeads (Miltenyi Biotec) and subjected to magnetic-activated cell sorting column (Miltenyi Biotec). The CD45-negative cells cultured for 2 days on plastic plates were used as cardiac myofibroblasts for further study.

For HSC isolation, the fibrotic livers of mice administered CCl_4_ for 4 to 6 weeks or fed a CDAHFD for 6 to 10 weeks were digested using 0.1% collagenase A (Roche), 0.015 mol/L HEPES (Nacalai Tesque), and Hanks' Balanced Salt Solution (Nacalai Tesque) at 37 °C for 40 min. The supernatant was then passed through a 70 μm filter and centrifuged at 50*g* for 1 min to remove hepatocytes. The supernatant subsequently obtained was collected and centrifuged at 300*g* for 5 min, and the pellets were treated with blood cell lysis buffer (Roche) for 1 min and seeded onto culture plates. The subsequent steps were the same as those used for cardiac myofibroblast isolation, and the CD45-negative cells obtained were used as activated HSCs for further analysis.

### Flow cytometry

The CD45-negative cells used as cardiac myofibroblasts and cultured for 2 days on plastic plates were disassociated from plastic plates *via* accutase (Nacalai Tesque) treatment. The cells were fixed with 1% paraformaldehyde (PFA; Nacalai Tesque) in PBS for 10 min and were subsequently permeabilized with 0.5% saponin for 10 min. After the permeabilization, the cells were stained with Alexa488-conjugated α-SMA (1:200; Invitrogen; 53–9760–82) antibody and analyzed using FACSAria (BD Biosciences).

### Cell culture and transfection

Mouse embryonic fibroblast cell line NIH3T3 cells were purchased from American Type Culture Collection. Human hepatic stellate cell line LX-2 cells were purchased from Merck Millipore. NIH3T3 cells, cardiac myofibroblasts, and activated HSCs were cultured at 37 °C with 5% CO_2_ in Dulbecco’s Modified Eagle’s Medium (DMEM; Nacalai Tesque) supplemented with 10% fetal bovine serum (FBS; Thermo Fisher Scientific) and 1% penicillin-streptomycin solution (Nacalai Tesque). LX-2 cells were cultured at 37 °C with 5% CO_2_ in DMEM supplemented with 2% FBS and 1% penicillin-streptomycin solution.

Lipofectamine RNAiMAX (Thermo Fisher Scientific) was used for siRNA transfection in NIH3T3 cells, LX-2 cells, cardiac myofibroblasts, and activated HSCs, as per the manufacturer’s instructions. For drebrin overexpression in NIH3T3 cells, Lipofectamine 2000 (Thermo Fisher Scientific) was used as per the manufacturer’s instructions.

### Treatment of NIH3T3 cells, cardiac myofibroblasts, and activated HSCs with TGF-β, CCG-1423, or Y27632

NIH3T3 cells were treated with 2 ng/ml recombinant TGF-β1 (R&D Systems) for 24 h. Cardiac myofibroblasts and activated HSCs were treated with 30 μM Y27632 (Wako) or 10 μM CCG-1423 (Sigma Aldrich) for 24 h after starvation in 0.1% FBS/DMEM for 24 h.

### Plasmid constructs and siRNAs

The cDNA encoding drebrin fused with FLAG or HA at the N terminus (FLAG-drebrin and HA-drebrin, respectively) was subcloned into pcDNA3. The SRF-RE Firefly Luc and Tk Renilla Luc plasmids were purchased from Promega, and 3 × FLAG MRTF-B was purchased from Addgene.

Silencer Select siRNAs were purchased from Life Technologies. The target sequence are as follows: mouse *Dbn1* (5′-GGUCCUAACUAUGCCCUCA-3′); mouse *Cthrc1* (5′-GGGAGAAUGCUUAAGGGAA-3′); mouse *Srf* (5′-AGAUGGAGUUCAUCGACAATT-3′); human *DBN1* (5′-CCACCUACCAGAAGACGGA-3′); Silencer Select Negative Control siRNA (Life Technologies) was used as a control.

### qRT-PCR

qRT-PCR was performed as previously described (23). The assay ID of Taqman probe purchased from Applied Biosystems is as follows:

mouse Mkl2: Mm00463877_m1.

The sequences of the primer pairs and dual-labeled probes purchased from Sigma-Aldrich are as follows:

mouse *Acta2*: 5′-CACCATGAAGATCAAGATCATTGCC-3′ (forward), 5′-GGTAGACAGCGAAGCCAGGA-3′ (reverse), and 5′-FAM-AGCCACCGATCCAGACAGAGTACTTGCG-TAMRA-3′ (probe);

human *Acta2*: 5′-GATCCTGACTGAGCGTGGC-3′ (forward), 5′-GGTAGACAGCGAAGCCAGGA-3′ (reverse), and 5′-FAM-ATTCCTTCGTTACTACTGCTGAGCGTGAGA-TAMRA-3′ (probe);

mouse *Col1a1*: 5′-CCCAAAGGTTCTCCTGGTGAAG-3′ (forward),

5′-CGGTTTTGCCATCAGGACCA-3′ (reverse),

and 5′-FAM-TGGTGCCAAGGGTCTCACTGGCAGTC-TAMRA-3′ (probe);

human *Colla1*: 5′-ACTGGTGACCTGCGTGTA-3′ (forward),

5′-GCCGCATACTCGAACTGG-3′ (reverse),

and 5′-FAM-CTCTCGCCAACCAGACATGCCTCTTG-TAMRA-3′ (probe);

mouse *Col3a1*: 5′-AAGCCCTGATGGTTCTCGAAA-3′ (forward),

5′-CTTGCAGCCTTGGTTAGGATC-3′ (reverse),

and 5′-FAM-ACCCAGTATTCTCCACTCTTGAGTTCGG-TAMRA-3′ (probe);

human *Col3a1*: 5′-CCCGAGGTGCTCCTGGTC-3′ (forward),

5′-CGATGTCCTTTGATGCCAGCA-3′ (reverse),

and 5′-FAM-CCACGTTCACCTGTTTCACCTTTGTCACCA-TAMRA-3′ (probe);

mouse *Cthrc1*: 5′-ATGCGCTCCAACAGTGCTC-3′ (forward),

5′-TGGTCCAGATAGATGATGGCTTC-3′ (reverse),

and 5′-FAM-TCTGTTCAGTGGCTCGCTTCGGCTCA-TAMRA-3′ (probe);

mouse *Dbn1*: 5′-CCTCCACCAACTCAAGAGGC-3′ (forward),

5′-GCTTTGCAACAGGGGAGGTT-3′ (reverse),

and 5′-FAM-CCCAGACCTCAGGAGCTGCTGTTACTTTG-TAMRA-3′ (probe);

human *Dbn1*: 5′-GGCCCTGGATGAGGTCACC-3′ (forward),

5′-GGCTCCTGGGTCTCTTGGG-3′ (reverse),

and 5′-FAM-CCTCCACCACTGCCACCGCCAC-TAMRA-3′ (probe);

mouse *Tagln*: 5′-TGACGAGGAGCTGGAGGAG-3′ (forward),

5′-ACAGGCTGTTCACCAATTTGC-3′ (reverse),

and 5′-FAM-AATCACACCATTCTTCAGCCACACCTGGAA-TAMRA-3′ (probe);

mouse *Gapdh*: 5′-CGTCCCGTAGACAAAATGGTGA-3′ (forward),

5′-CCACTTTGCCACTGCAAATGG-3′ (reverse),

and 5′-FAM-CCAATACGGCCAAATCCGTTCACACCGA-TAMRA-3′ (probe);

human *Gapdh*: 5′-TGGGCTACACTGAGCACCAG-3′ (forward),

5′-GCCAAATTCGTTGTCATACCAGG-3′ (reverse),

and 5′-FAM-TTCAACAGCGACACCCACTCCTCCACC-TAMRA-3′ (probe).

and Mouse *Mkl1*: 5′-CCCACATCTCTGCTGAAGAAGG-3' (forward),

5′-GACTGGAGGAGCCATTTTCCTG-3' (reverse),

and 5′-FAM-AGGCTGCTGGGTCACAGTCTCTTCATAAC-TAMRA-3' (probe).

The data for the target gene expression levels were normalized to those for the mouse or human *Gapdh* expression levels.

### Lysis and immunoprecipitation

The NIH3T3 cells were washed with PBS and lysed on ice in lysis buffer [50 mM Hepes-NaOH (pH 7.5)/150 mM NaCl/1% NP-40, 0.05% SDS/0.25% sodium deoxycholate/1 mM EGTA/1 mM MgCl2] supplemented with a protease inhibitor (1/100; Nacalai Tesque) and a phosphatase inhibitor (1/100; Nacalai Tesque). Then, the cell lysates were centrifuged at 15,000 rpm for 20 min at 4 °C; the supernatants were collected, added to 4 × SDS sample buffer, boiled at 95 °C for 5 min, and immunoblotted.

For collagen 1 samples, cardiac myofibroblasts were lysed in radioimmunoprecipitation assay buffer supplemented with a protease inhibitor (1/100; Nacalai Tesque), a phosphatase inhibitor (1/100; Nacalai Tesque), and 2-mercaptoethanol (1/100; Wako) at 20 °C for 20 min. The cell lysates were boiled at 95 °C for 10 min and centrifuged at 4 °C for 20 min at 15,000 rpm; the supernatants were collected and added to 4 × SDS sample buffer.

For nuclear extraction samples, cardiac myofibroblasts treated with siRNA were lysed using NE-PER Nuclear and Cytoplasmic Extraction Reagents (Thermo Fisher Scientific) according to the manufacturer’s protocol.

For immunoprecipitation, the supernatants collected were incubated overnight with an anti-FLAG magnetic antibody (Wako) on a rotary shaker at 4 °C. Next, they were washed with lysis buffer, following which the bound proteins were eluted with 2 × SDS sample buffer, boiled at 95 °C for 10 min, and collected as the immunoprecipitated samples.

### Immunoblotting

Immunoblotting was performed as previously described (23). The antibodies used in this study were as follows: mouse anti-GAPDH antibody (1:20,000; Santa Cruz; sc-32233), mouse anti-α-SMA antibody (1:10,000; Thermo; MS-113-P), rabbit anti-SM22α antibody (1:4000; Abcam; ab14106), rabbit anti-Col1a1 antibody (1:4,000, CST; 72,026), rabbit anti-β-actin antibody (1:4000; Proteintech; 20536-1-AP), rabbit anti-Mkl1 antibody (1:4000; Proteintech; 21166-1-AP), rabbit anti-lamin A/C antibody (1:4000; Proteintech; 10298-1-AP), rabbit anti-SOX9 antibody (1:4000; abcam; ab185230), horseradish peroxidase (HRP)-conjugated anti-FLAG primary antibody (1:10,000; Sigma Aldrich; A8592), horseradish peroxidase (HRP)- conjugated anti-HA primary antibody (1:5000; Roche; 12013819001), HRP-conjugated anti-mouse IgG secondary antibody (1:10,000; Abcam; ab6789), HRP-conjugated anti-rabbit IgG secondary antibody (1:10,000; Abcam; ab6721), HRP-conjugated anti-rabbit IgG secondary antibody (1:10,000; Santa Cruz; sc-2004), and HRP-conjugated anti-rabbit IgG secondary antibody (1:2000; CST; 7074).

### Immunocytochemistry

Cardiac myofibroblasts were fixed in 4% PFA(Nacalai Tesque) in PBS at 20 °C for 15 min and permeabilized in 0.1% Triton X-100 (Wako) in PBS at 20 °C for 5 min. Then, the cells were blocked at 20 °C for 1 h with blocking buffer containing 5% bovine serum albumin (BSA; Sigma Aldrich), 0.01% Triton X-100, and PBS and incubated at 4 °C overnight with rabbit anti-MRTF antibody (1:200; Proteintech; 21166-1-AP) diluted in antibody dilution buffer containing 5% BSA, 0.01% Triton X-100, and PBS. Subsequently, the cells were incubated for at 20 °C 1 h with Alexa594-conjugated donkey anti-rabbit IgG secondary antibody (1:200; Jackson; 711–585–152) diluted in antibody dilution buffer containing 5% BSA, 0.01% Triton X-100, and PBS. The cells were then washed with PBS and incubated at 20 °C for 5 min with 4′,6-diamidino-2-phenylindole (DAPI; 1:1,000, Dojindo) in PBS. They were subsequently mounted with FluorSave Reagent (Millipore) and observed using a confocal microscope (LSM700).

For phalloidin staining, fixation and permeation were performed using the same method, and the cells were incubated at 20 °C for 1 h before nuclear staining with iFluor488-conjugated phalloidin (1:1000; Abcam; ab176753) diluted with 1% BSA in PBS.

### *In situ* hybridization

For *Dbn1* mRNA detection in fibrotic hearts and livers, we used RNAscope Multiplex Fluorescent Reagent kit v2 (ACD) as per the manufacturer’s instructions. The fibrotic heart and liver were fixed with 4% PFA overnight, incubated in 20% sucrose (Nacalai Tesque), and embedded in optimal cutting temperature compound (Sakura Finetek). Next, the heart and liver samples were sectioned into 6 and 10 μm thick sections, respectively, and used for RNAscope analysis

For performing Tnni3 or Desmin costaining after staining for *Dbn1* mRNA, the sections were blocked with 10% BSA in PBS for 1 h and incubated at 4 °C overnight with goat anti-Tnni3 antibody (1:200; Abcam; ab56357) or rabbit anti-Desmin antibody (1:200; Abcam; ab32362) diluted with 10% BSA in PBS. Then, the cells were incubated at 20 °C for 1 h with Alexa488-conjugated donkey anti-goat IgG secondary antibody (1:200; Abcam; ab150129) or Alexa488-conjugated donkey anti-rabbit IgG secondary antibody (1:200; Invitrogen; A21206) diluted with 10% BSA in PBS. The cells were then washed with PBS and incubated with 4′,6-diamidino-2-phenylindole (1:1000; Dojindo) in PBS at 20 °C for 5 min, following which they were mounted with FluorSave reagent and observed using a confocal microscope (LSM700).

### Luciferase assay

The luciferase assay was performed using the Dual-Luciferase Reporter Assay System (Promega) as per the manufacturer’s instructions. Briefly, NIH3T3 cells transfected with plasmids encoding drebrin and luciferase were washed with PBS and lysed in passive lysis buffer. Luminescence was detected using the EnSpire system (PerkinElmer).

### RNA-seq analysis

The total RNA samples extracted in this study were sent to Macrogen Japan for library construction, sequencing, and read mapping. Briefly, libraries were prepared using the TruSeq Stranded mRNA LT Sample Prep Kit (Illumina) after quality control and were subsequently sequenced on an Illumina NovaSeq 6000 system. Adaptor removal and quality trimming were performed using Trimmomatic, and the trimmed reads were aligned to the mouse reference genome (mm10) by using HISAT2. StringTie was used for transcript assembly, the read count was calculated, and the values obtained were normalized to the fragments per kilo base per million mapped reads and transcript per million (TPM) values. The read counts and fragments per kilo base per million mapped reads and TPM values have been provided in Table S1. MA plots were created on the basis of the TPM values.

The datasets of human patients with cardiac fibrosis (GSE116250) and hepatic fibrosis (GSE162694) were downloaded from the Gene Expression Omnibus (GEO) database. Target gene expression was analyzed after the raw counts were normalized.

### KEGG pathway analysis

The genes showing downregulation after siDbn1 treatment (M < −1.0, A > 0) were analyzed using DAVID Bioinformatics Resources version 6.8. The clusters obtained helped identify KEGG pathways with gene counts greater than 25.

### Bioinformatics analysis of single-cell data

The single-cell dataset of mouse cardiac interstitial cells at 3 and 7 days after sham operation or MI was downloaded from ArrayExpress under accession E-MTAB-7376 and that of mouse liver cells treated with corn oil or CCl_4_ was downloaded from GEO under accession GSE171904. The data were processed using the Seurat package. We removed all cells with fewer than 200 genes, and all genes were expressed in fewer than three cells. In addition, cells with more than 10% or 20% mitochondrial genes were filtered out. Subsequently, gene expression measurements were normalized, and 2000 variable genes were selected for dimensionality reduction. The data were then scaled, and linear dimensional reduction was performed. The cells were then clustered and visualized using UMAP. Genes that were differentially expressed between the two groups were identified using the Seurat package, and a heat map was generated using the ggplot2 package. The gene abundance in *Dbn1*-positive cells (log2 (fold change) > 0.2) was analyzed using DAVID Bioinformatics Resources, version 6.8.

### Statistical analysis

All results are provided in terms of mean ± standard deviation (SD) values. Statistical analysis was performed using the two-tailed unpaired Student’s *t* test for comparisons between two groups or one-way analysis of variance with Tukey’s range test for multigroup comparisons by using GraphPad Prism 5.0. Statistical significance was set at *p* < 0.05.

## Data availability

The RNA-Seq data generated in this study are available in the Supporting Information. Publicly available RNA-Seq datasets of human patients with tissue fibrosis were retrieved from the GEO database under accession GSE116250 (heart) or GSE162694 (liver). Publicly available single-cell datasets of mouse cardiac cells and mouse liver cells were obtained from ArrayExpress under accession E-MTAB-7376 and from the GEO database under accession GSE171904. All the R scripts used in this study are available from the authors upon reasonable request.

## Supporting information

This article contains supporting information.

## Conflict of interest

All authors declare no conflicts of interest with the contents of this article.
