# Supplementary material for: The well-developed actin cytoskeleton and Cthrc1 expression by actin-binding protein drebrin in myofibroblasts promote cardiac and hepatic fibrosis
Source: J Biol Chem. 2023 Jan 20;299(3):102934. doi: 10.1016/j.jbc.2023.102934 (PMC9988570; doi:10.1016/j.jbc.2023.102934)
Supplement: Supplemental Figure 1-4 [file mmc2.pdf]

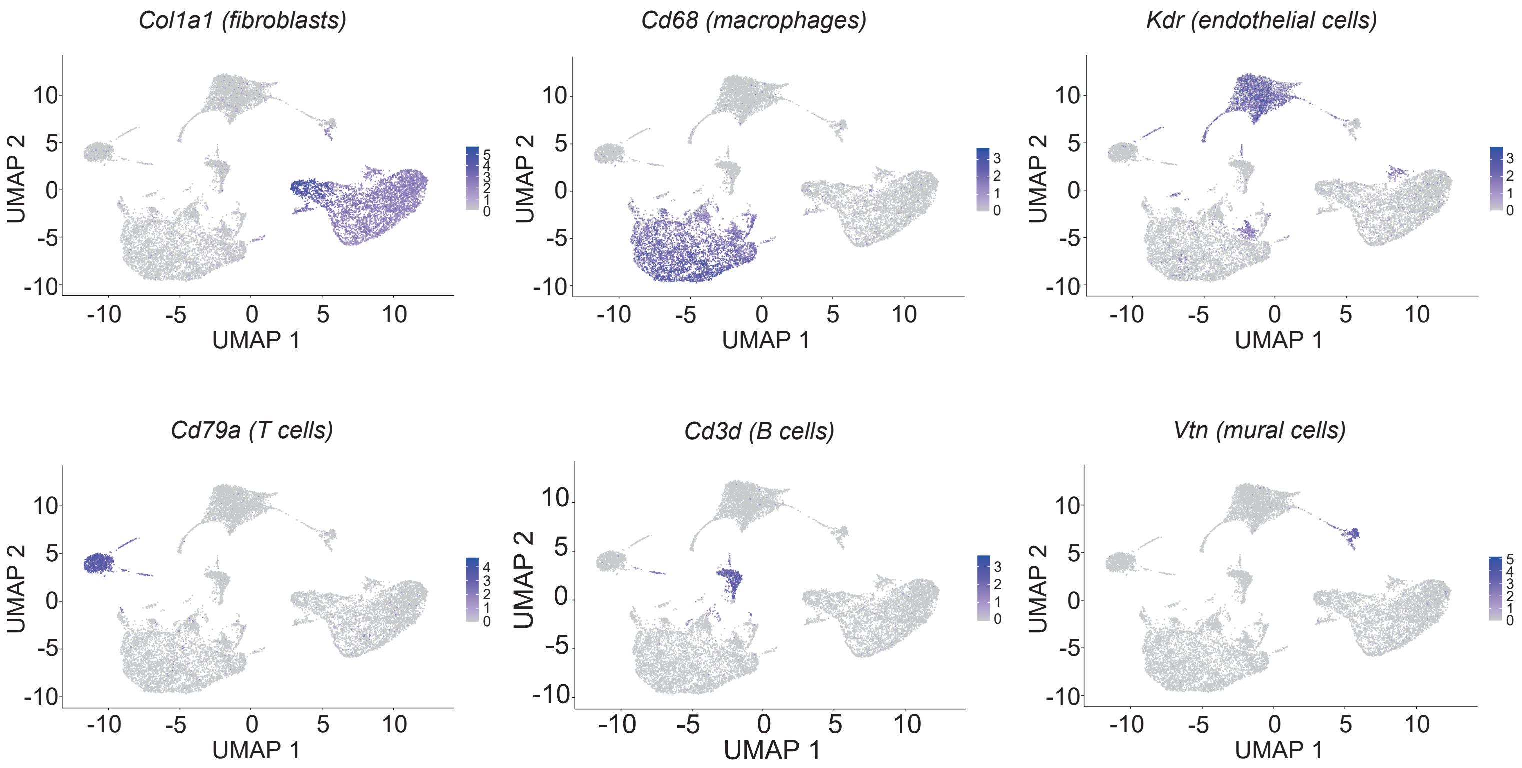

Fig. S1 The mRNA expression levels of *Col1a1*, *Cd68*, *Kdr*, *Cd79a*, *Cd3d*, and *Vtn* in mouse cardiac interstitial cells 3 or 7 days after sham or MI. The publicly available data (E-MTAB-7376) were reanalyzed using R package Seurat.

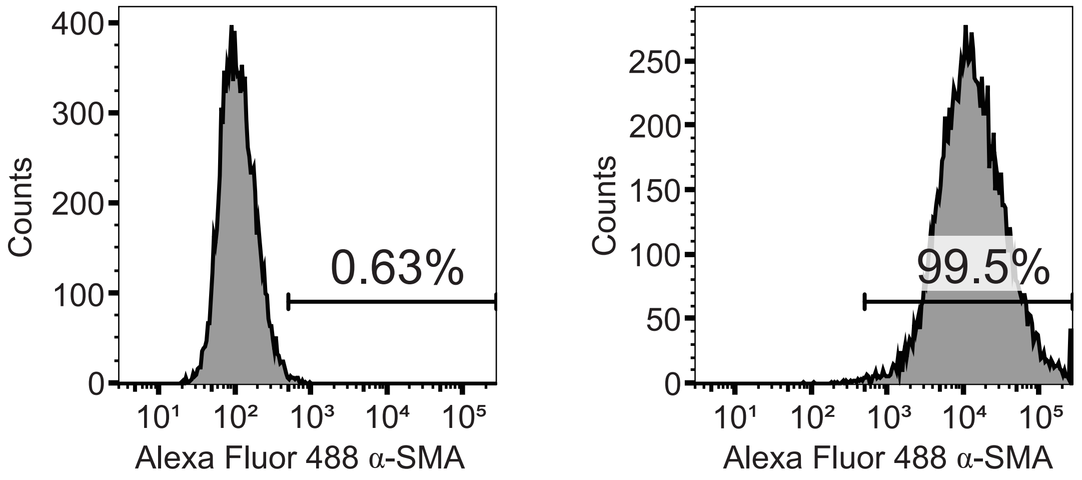

Fig. S2 The α-SMA protein expression levels in cells, which we used as cardiac myofibroblasts in our in vitro experiments. CD45-negative cells were isolated from infarcted mouse hearts and cultured on plates for 2 days. The cells were stained with anti-α-SMA antibody, and subsequently analyzed by flowcytometry.

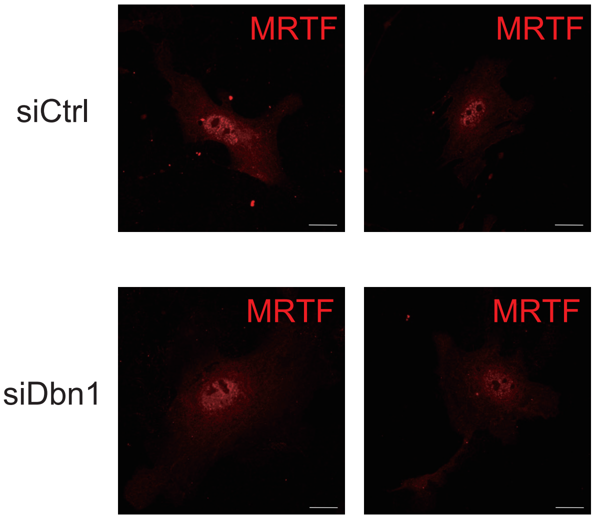

Fig. S3 MRTF translocation in cardiac myofibroblasts treated with siCtrl or siDbn1. At 48 h after siRNA transfection, the cells were seeded onto glass-bottom dishes, cultured for 24 h, and subjected to immunocytochemistry. Scale bar: 20 µm.

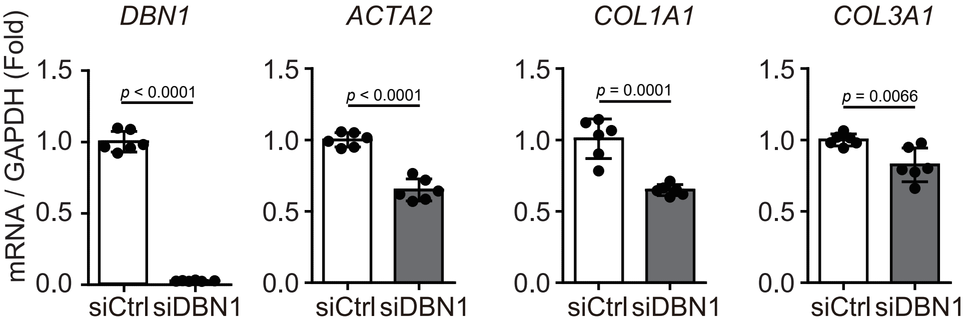

Fig. S4. The mRNA expression levels of fibrosis-related genes in LX-2 cells treated with siCtrl or siDBN1. At 72 h after the transfection of siRNA, LX-2 cells were lysed and subjected to qRT-PCR. n = 6.
